# Sister Mary Joseph's Nodule as a First Manifestation of Primary Peritoneal Cancer

**DOI:** 10.1155/2012/467240

**Published:** 2012-10-24

**Authors:** C. Iavazzo, K. Madhuri, S. Essapen, N. Akrivos, A. Tailor, S. Butler-Manuel

**Affiliations:** Department of Gynaecological Oncology, Royal Surrey County Hospital, Guildford, Surrey GU2 7XX, UK

## Abstract

Sister Mary Joseph's nodule metastasis is a rather rare finding. The primary malignancy in women is usually ovarian, endometrial, gastric, or pancreatobiliary tree cancer. We present a case of an 87-year-old patient with Sister Mary Joseph's nodule metastasis caused by a primary peritoneal malignancy. Through a literature search, we also discuss the pathophysiology, diagnostic approach, management, and prognosis of such a condition.

## 1. Introduction

Sister Mary Joseph's nodule could be the first sign of internal malignancy which is usually found in the gastrointestinal tract (52%), female genital tract or genitourinary tract (28%), and nonspecified origin (18–20%) [1]. The majority of them are of gastric or ovarian origin; however, cases from appendix, gallbladder, pancreatic, hepatobiliary, or endometrial carcinomas are also described in the literature [2–4]. When such a nodule is identified, it suggests not only the presence of a malignancy, but also poor prognosis. 

Sister Mary Joseph whose name was Julia Dempsey (1856–1939) was working as a superintendent nurse at St. Mary's Hospital in Rochester, Minnesota, when she identified that an umbilical nodule could be a sign of intra-abdominal or pelvic malignancy. Sister Mary Joseph was the first surgical assistant under the guidance of Dr. William Mayo who first reported the condition as “pants button umbilicus” in 1928. The eponym was coined to her by Hamilton Bailey in 1949 in his book entitled “Demonstration of Physical Signs in Clinical Surgery” [5].

The aim of this study is to present a case of a patient with Sister Mary Joseph's nodule and discuss the process from diagnosis to management, as well as the possible mechanisms of such a metastasis, differential diagnosis, and prognosis.

## 2. Case

**Figure 1:**
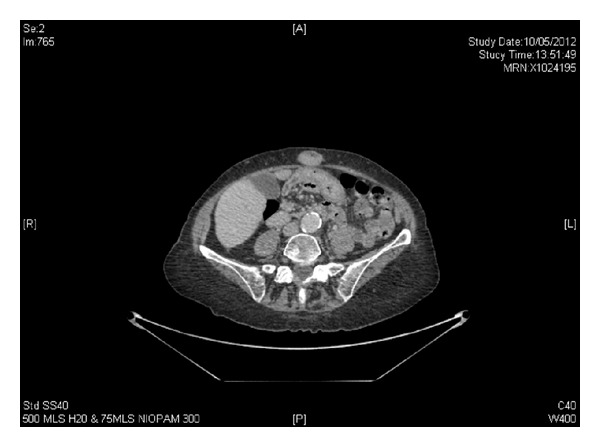
Sister Mary Joseph's nodule.

This is a case of an 87-year-old patient who presented in our department with Sister Mary Joseph's nodule. The patient had a history of hypertension, hyperthyroidism, osteoporosis, and aortic aneurysm. The patient referred that she had appetite loss, vomiting, and weight loss. The physical examination revealed distended abdomen. Biopsies taken from the umbilical nodule under ultrasound guidance demonstrated a serous carcinoma of primary peritoneal/ovarian origin. Regarding tumor markers, CA 125 levels were 50 ku/L, CA 15-3 10 ku/L, and CA 19-9 <2 ku/L. The CT scan measured the umbilical node to be 21 × 22 mm (Figure 1). Because of her medical history, the patient received palliative radiotherapy to umbilical nodule (16 Gy in two fractions) and then commenced tamoxifen. The nodule reduced in size and the patient was followed up every three months with clinical examination, imaging and tumor markers. After one and a half year from the first diagnosis, the umbilical nodule measured approximately 2-3 cm on clinical examination, CA125 levels were raised, and the CT scan revealed that Sister Mary Joseph's nodule was larger (2.5 cm), while there was also a small presacral nodule on the left side as well as a nodule overlying the psoas muscle. The patient underwent wide excision of the umbilical nodule because of the malodor and size increase. Histology revealed a serous carcinoma metastasis of primary peritoneal origin. 

## 3. Discussion

It is estimated that 1–3% of patients with abdominopelvic malignancy could present with a Sister Mary Joseph's nodule [1]. Epidemiological studies revealed that this condition predominates in women [1]. 

The possible mechanism of tumor spread to Sister Mary Joseph's nodule could be through lymph ducts, blood vessels, contiguous extension, or embryologic remnants in the anterior abdominal wall [6]. More specifically, the umbilical region is connected by the lymphatic system to the axillary, inguinal, and para-aortic lymph nodes. After birth, the fetal cord structures develop into ligaments or peritoneal folds. On the lateral umbilical folds, the inferior epigastric vessels and, sometimes, a vestigial vitelline duct connecting the umbilicus to the ileum can be recognized. Moreover, the umbilical region shows a rich arterial supply that includes the inferior epigastric and deep circumflex iliac branches of the external iliac artery and the superior epigastric branch of the internal mammary artery. Regarding venous supply, several anastomotic branches exist from the internal mammary vein or through the superficial epigastric vein or from the portal system of the liver. The dissemination of malignant cells through the urachus is another possible mechanism. Periumbilical skin is unique due to its proximity to intra-abdominal and pelvic structures; for this reason contiguous extension could be suggested [6].

According to a retrospective study including 77 cases of umbilical malignancies, 88% of such cases originated outside the umbilicus. From these, 85% patients with metastatic tumors had a recognized primary malignancy. In women, the three most common primary sites were found to be the ovary, the endometrium, and the pancreatobiliary tree [7]. The mean age of diagnosis of cases with Sister Mary Joseph's nodule is 50.6 years (range 18–87 years) [8, 9]. According to our knowledge, our case is the oldest in the current literature. 

Sister Mary Joseph's nodule is usually located several centimeters away from the possible omental disease. It can be seen or felt deep in the subcutis in the umbilical area. Incidental detection of such a metastasis for umbilical hernia repair is also described [10]. It could be painful and ulcerated. It is usually described as a painful nodule with irregular margins, hard or even necrotic. The nodule could reach 10 cm in diameter, but the mean diameter is 2-3 cm, while 75% of them are adenocarcinomas [1, 6]. In our patient, the nodule was the first manifestation of a stage IIIC primary peritoneal carcinoma.

Similar cases are described in the literature. Kurt et al. described a case of Sister Mary Joseph's nodule as a first sign of a stage IV mixed-type epithelial ovarian carcinoma which was the first case with serous component [11]. Moreover, such a nodule is described even in cases with micropapillary serous ovarian carcinomas [12].

Fine needle aspiration cytology and core biopsy are proposed as simple, fast, accurate, and inexpensive diagnostic tools. Handa et al. using this method identified primary carcinoma of the stomach in three cases, ovary in two cases and one case was of non-Hodgkin's lymphoma during a 5-year period [13]. Moreover, histological and immunohistochemical examination could lead to the diagnosis. The differential diagnosis includes umbilical hernia, cutaneous endometriosis, lymphangioma, melanoma, pilonidal sinus and pyogenic granuloma [1–6].

Dermatologists, general surgeons, gynaecologists, and general practitioners should be aware of such a relatively rare entity. Aggressive treatment is proposed combining surgical excision, radiotherapy, and chemotherapy; however as the disease is usually advanced and metastatic, only palliative treatment is offered. After multidisciplinary team decision and informed consent, our patient had palliative radiotherapy only, but the tumor persisted and increased in size. Surgical excision was decided in a day-case basis which was very well tolerated despite the coexisting very significant comorbidity. The mean patient's survival is 10–12 months [1]. It should be mentioned that the survival is better in patients who detected such a metastasis before definitive treatment of the primary tumor rather than as a recurrence of it [6]. Survival could reach 17.6 months if wide excision is combined with radiotherapy and chemotherapy. Less than 15% of the patients survive for over 2 years depending on the primary malignancy and response to palliative treatment [1, 6, 11, 13].

## 4. Conclusion

Careful examination combined with fine needle aspiration or biopsy is proposed to further clarify the diagnosis of a Sister Mary Joseph's nodule. Although the prognosis is not favorable, palliative treatment is usually proposed.
